# Extracellular Regucalcin: A Potent Suppressor in the Cancer Cell Microenvironment

**DOI:** 10.3390/cancers17020240

**Published:** 2025-01-13

**Authors:** Masayoshi Yamaguchi

**Affiliations:** Cancer Biology Program, University of Hawaii Cancer Center, University of Hawaii at Manoa, 701 Ilalo Street, Honolulu, HI 96813, USA; yamamasa11555@yahoo.co.jp or myamaguchi@cc.hawaii.edu

**Keywords:** regucalcin, cancer suppressor, cancer microenvironment, biomarkers

## Abstract

The regucalcin gene is located on the X chromosome, comprising seven exons and six introns. This gene and protein are expressed in various tissues and cells and is predominantly expressed in human liver, kidney, and adrenal tissues. Regucalcin gene expression is enhanced via a mechanism mediated by several signaling molecules and transcription factors. Regucalcin plays a multifunctional role in cellular regulation in maintaining cell homeostasis. In addition, regucalcin has been implicated in several metabolic disorders and diseases. In particular, regucalcin plays a role as a novel suppressor in several types of cancer patients. Increased expression of regucalcin suppresses the growth of human cancer cells, suggesting its pivotal role in suppressing tumor development. The survival time of cancer patients is prolonged with increased expression of regucalcin in the tumor tissues. The adhesion, migration, invasion, and bone metastatic activity of cancer cells are blocked by the overexpression of regucalcin, promoting dormancy in cancer patients. Interestingly, regucalcin is also found in human serum, suggesting its character as a novel biomarker in various diseases. This extracellular regucalcin has been shown to suppress human cancer cells’ growth and bone metastatic activity. Thus, extracellular regucalcin may play a vital role as a suppressor of human cancer activity. Alteration of the serum regucalcin levels in physiological and pathophysiological conditions may influence the activity of cancer cells in the microenvironment. This review will discuss the potential role of extracellular regucalcin in cancer cell activity as a critical suppressor in the cancer microenvironment.

## Simple Summary

Regucalcin plays a multifunctional role in cellular regulation in maintaining cell homeostasis and has been implicated in several metabolic disorders and diseases. In particular, regucalcin is a novel suppressor in several cancer types. Regucalcin plays a potential role in suppressing carcinogenesis. The survival time of cancer patients is prolonged with increased expression of regucalcin in the tumor tissues. The migration, invasion, and bone metastatic activity of cancer cells are blocked by the overexpression of regucalcin, thereby promoting dormancy in cancer patients. Notably, regucalcin is found in serum and extracellular fluids. Extracellular regucalcin has been shown to suppress the growth and bone metastatic activity of human cancer cells, suggesting that it plays an important role as a suppressor in the tumor microenvironment of cancer cells. Alteration of serum regucalcin levels under physiological and pathophysiological conditions may influence cancer activity in the microenvironment. This review discusses the potential role of extracellular regucalcin in cancer cell activity as a potential suppressor in the cancer microenvironment, proposing the role of exogenous regucalcin in cancer prevention and therapy.

## 1. Introduction

The classical intracellular signaling factors, including calcium and cyclic adenosine 3′,5′-monophosphate, play a pivotal role in the regulation of cell function by peptide hormones [1,2,3,4,5,6]. The regucalcin gene is located on the X chromosome, comprising seven exons and six introns. Regucalcin was discovered as a novel calcium-binding protein lacking the calcium-binding motif of the EF-hand structure in 1978 [7,8,9,10,11,12,13,14,15]. Regucalcin plays a crucial role as an inhibitory protein in calcium signaling, suppressing the activity of various enzymes, including protein kinase C [8], which are activated by Ca^2+^ or Ca^2+^/calmodulin [8]. Regucalcin gene expression is regulated by various transcription factors, including AP-1, NF1-A1, RGPR-p117, β-catenin, SP1, and others, which are identified as enhancers and suppressors, and this expression regulates one’s physiological state and is implicated in hormonal stimulation [16,17,18,19,20]. Regucalcin plays a multifunctional role in various cell functions, including cell calcium homeostasis and signaling mechanism, enzyme activity regulation, post-translational protein output, nuclear DNA and RNA synthesis, gene expression, cell proliferation, apoptotic cell death, energy metabolism, neuronal function, cardiovascular regulation, and testis formation [21,22,23,24,25,26,27,28,29,30]. In addition, regucalcin has been implicated in several metabolic disorders and diseases [21,30,31,32].

Notably, regucalcin has been shown to play a critical role as a novel suppressor in various cancer patients [33,34]. The growth of various human cancer cells with different types is blocked by the overexpression of regucalcin [35,36,37,38,39,40,41], suggesting its role as a suppressor of carcinogenesis. Interestingly, the survival of patients with various types of cancer is prolonged with increased expression of regucalcin in the tumor tissues [36,37,38,39,40,41]. The overexpression of regucalcin inhibits the cancer cells’ metastatic activity, including adhesion, invasion, migration, and bone metastasis [41]. Regucalcin promotes dormancy in cancer patients [34], with higher levels of regucalcin confirmed to be associated with longer recurrence-free and overall survival of prostate cancer patients. Thus, regucalcin can be a novel tumor suppressor in human cancer.

Interestingly, intracellular regucalcin is released into the serum from human and animal tissues, and its serum levels are altered by physiological and pathophysiological conditions [42,43,44,45], suggesting its role as a novel biomarker in various diseases [45]. However, the role of extracellular regucalcin in cell regulation remains to be elucidated. In recent years, increasing studies have indicated that extracellular regucalcin inhibits the growth of various types of human cancer cells [34], suggesting its suppressive role in the cancer microenvironment. Extracellular regucalcin may block the metastatic activity of human cancer cells, thus playing a potential role as a suppressor of human cancer. This study may provide new insights into cancer regulation and therapy.

This review outlines the role of intracellular regucalcin in cell regulation, signaling systems, and cell growth repression, and it discusses the inhibitory role of extracellular regucalcin on the cancer activity in various human cancer cells. This study proposes the involvement of extracellular regucalcin as a suppressor of cancer activity in the microenvironment.

## 2. Intracellular Regucalcin Regulates Several Signaling Pathways

Intracellular signaling factors, including calcium ions (Ca^2+^), cyclic 3′,5′-adenosine monophosphate (cyclic AMP), and nitric oxide (NO), play a central role as modulators of the hormonal regulation of cell function [1,2,3,4,5,6,46,47,48,49,50,51]. The effects of intracellular Ca^2+^ are modulated by Ca^2+^/calmodulin-dependent protein kinase and protein kinase C. Intracellular cyclic AMP is generated by peptide hormones, and this molecule is degraded by cyclic AMP phosphodiesterase. Ca^2+^/calmodulin activates this enzyme’s activity. Intracellular NO, which is known as a modulator molecule, is produced by NO synthase, which requires Ca^2+^/calmodulin for its activation [50,51,52]. These enzymic activities are inhibited by regucalcin. In addition, protein phosphorylation–dephosphorylation plays a crucial role in intracellular signal transduction. Calcineurin, a calmodulin-binding protein, is a Ca^2+^/calmodulin-dependent protein phosphatase [53]. Regucalcin inhibits the activities of calcineurin, protein tyrosine phosphatase, and protein serine/threonine phosphatases in the cytoplasm and the nucleus of cells [54]. Thus, regucalcin has been shown to play a crucial role in the regulation of several key enzymes associated with the intracellular signaling pathways. In addition, regucalcin is involved in the regulation of protein production in cells. Regucalcin has a suppressive effect on protein synthesis by inhibiting the activity of aminoacyl-tRNA synthetase, which is a rate-limiting enzyme in the translational process of protein synthesis by binding aminoacyl(leucyl)-tRNA synthetase [55]. In addition, regucalcin has been shown to activate cysteinyl proteases, including Ca^2+^-activated proteases (calpains), in cells [56,57]. Regucalcin may play a role in protein turnover regulation by inhibiting protein synthesis and activating protein degradation in cells.

Of note, regucalcin has been shown to regulate nuclear signaling systems. Cytoplasmic regucalcin is translocated to the nuclei and binds to nuclear calmodulin [58,59], which stimulates nuclear DNA synthesis [60,61]. The nuclear translocation of regucalcin is promoted by the signaling mechanism of protein kinase C in cells [62]. Nuclear DNA endonuclease is responsible for nuclear DNA fragmentation, leading to extensive Ca^2+^-dependent DNA hydrolysis, which induces apoptotic cell death [63]. Regucalcin inhibits nuclear Ca^2+^-activated DNA fragmentation [64]. Regucalcin protects against apoptotic cell death. Thus, regucalcin plays a regulatory role in nuclear signaling systems in cells. The signaling process from the cytoplasm to the nucleus is regulated by various protein kinases and protein phosphatases involved in gene expression and cell proliferation. Regucalcin has been shown to inhibit the activities of Ca^2+^-dependent protein kinases, protein phosphatases, and DNA [65,66] and RNA [67,68] synthesis in the nucleus. Regucalcin has been shown to bind directly to DNA [58,59]. Regucalcin may play a potential role in the transcription process in the cell nucleus [24].

As described above, regucalcin plays a suppressive role in signal transduction from the cytoplasm to the nucleus in cell regulation, which is mediated by the phosphorylation and dephosphorylation of various cell proteins. Intracellular regucalcin plays a pivotal role as a regulator of the intracellular signaling systems.

## 3. The Suppressive Role of Intracellular Regucalcin in Cell Growth

Regucalcin mRNA is expressed in liver cancer cells at lower levels [69,70]. Regucalcin has been shown to suppress enhancements in cell proliferation, although it does not stimulate apoptotic cell death. The overexpression of regucalcin protects against apoptotic cell death induced by various factors [26,71,72], and it has been shown to block cell proliferation by inducing G1- and G2/M-phase cell cycle arrest [73]. The suppressive effect of regucalcin on cell proliferation is mediated through inhibition of the activities of Ca^2+^ signaling-dependent protein kinases and PI3 kinase [25,33,34,73]. Furthermore, the suppressive effects of regucalcin on cell proliferation are mediated by the inhibition of cascades of various protein kinases [74] and protein phosphatases [75]. Endogenous regucalcin has been shown to inhibit the increased protein phosphatase activity associated with cell proliferation, and this increase is blocked by adding regucalcin antibodies into the enzyme reaction mixture [76,77]. Notably, regucalcin suppresses the enhancement of DNA synthesis in the nuclei of cancer cells associated with cell proliferation [77,78]. The suppressive effects of regucalcin on cell proliferation are based on the same mechanism in normal and cancer cells.

Regucalcin causes cell cycle arrest in the G1 and G2/M phases in cancer cells [25]. Regucalcin has been shown to suppress the gene expression of cell-cycle-related proteins in proliferating cancer cells [25,79], including cdc2, cdk2m, chk2 (checkpoint kinase 2), and p21 mRNAs [80,81,82,83,84]. The overexpression of regucalcin suppressed the expression of IGF-I mRNA in liver cancer cells [79], resulting in a delay in cell proliferation. In addition, regucalcin regulates the gene expression of tumor-related proteins, including the tumor stimulator genes c-*myc,* c-*fos*, c-*jun,* and Ha-*ras* [25,33,34,79], the tumor suppressor genes *p53* and *Rb*, and the oncogene *c-src* [83,84,85]. Alterations in these genes’ expression are reversed by the overexpression of regucalcin in cancer cells [25,33,34], resulting in cell growth retardation. Downregulation of regucalcin gene expression in cancer cells may lead to stimulation of cell proliferation with alterations in the expression of various tumorigenesis-related genes.

Notably, regucalcin has been shown to alter growth in an in vivo regenerating liver model. Adult rat hepatocytes are normally quiescent in vivo. Partial hepatectomy induces a synchronous wave of DNA synthesis and cell division, with the cells continuing to divide until the original mass of the liver is regenerated [86,87,88,89]. Hepatocyte growth factor, which promotes liver regeneration after partial hepatectomy, has been shown to stimulate Ca^2+^/calmodulin, nuclear protein kinase, and protein phosphatase activities in liver generation [87,88,89]. The regucalcin mRNA expression in the regenerating liver is stimulated by Ca^2+^/calmodulin in the liver [90,91,92,93]. The overexpression of regucalcin has been shown to suppress the enhancement of calcium signaling and protein, DNA, and RNA synthesis in the cytoplasm and nuclei of regenerating liver cells in vivo [94,95,96,97]. Increased regucalcin may have contributed to the suppression of cell proliferation in regenerating rat livers in vivo [94,95,96,97]. Thus, regucalcin may suppress the overproliferation of cells in vivo.

## 4. The Role of Intracellular Regucalcin as a Suppressor of Human Cancer

Regucalcin is a potential suppressor of several types of human cancer [25,32,33,34]. Regucalcin gene and protein expression is downregulated in physiological and pathophysiological conditions [42]. The overexpression of regucalcin has been shown to suppress the growth of cancer cells by inhibiting the intracellular signaling pathways associated with cell proliferation, including suppression of oncogene levels and stimulation of tumor suppressor genes in cancer cells [34,35,36,37,38,39,40,41,98,99]. Increased expression of regucalcin in the tumor tissue prolongs the survival of cancer patients [34,35,36,37,38,39,40,41]. Intracellular regucalcin plays a critical role as a suppressor in human cancer. Its downregulation may lead to tumor promotion [42].

Regucalcin is greatly expressed in human livers. Regucalcin expression is downregulated in human liver cancer. Hepatocellular carcinoma (HCC) is the most common primary liver cancer [100,101]. Tissue microarrays have confirmed that the levels of regucalcin are decreased in HCC tissue compared to those in non-tumor tissue [35,102]. Higher expression of the regucalcin gene was associated with prolonged survival in HCC patients in comparing the clinical evaluation of 81 HCC patients with higher regucalcin levels and 81 HCC patients with lower regucalcin levels [34,35]. The overexpression of regucalcin blocked the G1 and G2/M phases of the cell cycle and the proliferation of human liver cancer cells by inhibiting several signaling pathways, including calcium, Ras, Akt, MAP kinase, SAPK/JNK, NF-κB p65, β-catenin, c-fos, and c-myc signaling, while increasing p21, p53, and Rb [34]. Regucalcin downregulation in HCC may be involved in DNA methylation [102].

Human lung cancer is divided into two types: small-cell lung cancer (SCLC) and non-small-cell lung cancer (NSCLC). NSCLC is related to malignancy-related deaths [103,104,105,106,107,108,109]. Epigenetic modifications of survivin and regucalcin in NSCLC tissues contribute to malignancy [110]. The GEO database of 204 patients with lung adenocarcinoma showed that regucalcin was decreased in the patients with lung cancer, and a higher regucalcin gene expression led to prolonged survival in lung cancer patients [38]. Additionally, a Kaplan–Meier survival analysis of tumor and normal tissues from 341 NSCLC patients showed that patients with lower regucalcin levels experienced worse overall survival in comparison with that of patients with a higher regucalcin expression [111]. The overexpression of regucalcin suppressed proliferation and migration in human NSCLC A549 cells in vitro by suppressing several signaling pathways [38].

Prostate cancer is one of the most common malignancies and is the second leading cause of cancer-related death in men [112,113,114,115]. Prostate cancer patients with higher levels of regucalcin showed a longer progression-free survival than those with a lower regucalcin gene expression [41]. Prostate cancer patients with a higher regucalcin expression showed a longer recurrence-free and overall survival. The overexpression of regucalcin was found to suppress the migration and invasion of bone metastatic human prostate cancer cells in vitro by reducing the levels of key metastatic proteins, including Ras, Akt, MAP kinase, RSK-2, mTOR, caveolin-1, and integrin β1 [116]. In addition, knockdown of regucalcin in the LNCap human prostate cancer cells resulted in an increase in the growth of mice’s tibias [117].

Breast cancer is prone to metastasize to the bone, causing distressing pathologic fractures, pain, and hypercalcemia [118,119,120,121,122,123,124,125,126]. In human breast cancer patients, the clinical outcomes between 44 patients expressing higher levels of regucalcin and 43 patients with a lower expression were compared by evaluating data from the GEO database (GSE6532) [37]. Regucalcin expression was found to be downregulated in the patients with breast cancer, and patients with higher levels of regucalcin in the tumor tissue had a longer recurrence-free survival [37]. The overexpression of regucalcin induced arrest of the cell cycle and suppressed human breast triple-negative MDA-MB-231 cell proliferation by suppressing various signaling pathways, including Akt, MAP kinase, SAPK/JNK, NF-κB p65, and β-catenin pathways [37].

Pancreatic ductal adenocarcinoma (PDAC) comprises approximately 90% of pancreatic cancers [127,128,129]. Regucalcin is shown to be a tumor suppressor in human pancreatic cancer [36]. A microarray analysis showed a decrease in the regucalcin levels in the pancreatic tissue from 36 PDAC patients as compared to tissue from 36 normal pancreases [34]. The survival of pancreatic cancer patients associated with an increase in regucalcin gene expression was prolonged [36]. The overexpression of regucalcin suppressed the proliferation and migration of K-ras mutated MIA PaCa-2 human pancreatic cancer cells showing resistance to drug therapy and radiotherapy [36]. Overexpressed regucalcin blocked several signaling pathways, including Akt, MAP kinase, SAPK/JNK, K-ras, c-fos, and c-jun pathways, and increasing the levels of the tumor suppressor p53 [36].

Adenocarcinoma colorectal cancer (CRC) is a commonly diagnosed cancer [130,131,132]. Even with the development of new therapeutic strategies, the prognosis for CRC remains poor [133,134,135,136]. The KRAS gene mutation is found in many tumors [137,138,139,140]. Survival data for 62 patients from the GEO database (GSE12945) showed that the expression of regucalcin was reduced in CRC patients [39]. The survival of colorectal cancer patients is prolonged with increased regucalcin gene expression in their tumor tissues [39]. The overexpression of regucalcin suppressed the colony formation and proliferation of human CRC-derived RKO cells by inhibiting various signaling pathways [39].

Renal cell carcinoma (RCC) is the most common cause of death among urological malignancies [141,142,143,144,145,146]. Agents approved by the FDA for clear-cell RCC are agents including the mammalian target of rapamycin (mTOR), vascular endothelial growth factor (VEGF), platelet-derived growth factor (PDGF), and their respective receptors [147,148]. These inhibitors may be limited by the initiation of a drug-resistant phenotype [149,150,151,152,153]. The regucalcin expression is downregulated in RCC tumor tissue [40]. Prolonged survival was observed in RCC patients with a higher regucalcin gene expression [40]. The overexpression of regucalcin suppressed A498 cell proliferation by inhibiting the expression of several intracellular signaling molecules [40].

Cervical cancer involves tumors with a high rate of ovarian metastasis [154,155,156,157,158,159,160,161]. Lentivirus-mediated regucalcin transfected into HeLa cells increased their regucalcin expression and suppressed cell proliferation and metastatic activity, resulting in reduced levels of β-catenin, p-glycogen synthase kinase-3β (GSK-3β), and matrix metalloproteinases (MMPs)-3, -7, and -9 [162,163]. Of note, downregulated regucalcin expression was shown to stimulate cell proliferation and metastatic activity [163].

Melanoma is a highly aggressive form of skin cancer [164,165]. Breslow’s thickness (T stage) [166] is one of the key factors in determining the prognosis and treatment of locally advanced melanoma and is based on the thickness of the main tumor in millimeters. Biomarkers may be associated with the clinical stage and tumor progression [167]. An affinity proteomics analysis of 149 serum samples from melanoma patients showed lower serum regucalcin and syntaxin 7 levels as compared with these levels in those with no recurrence [167]. Regucalcin may be an important biomarker in human melanoma.

Chondrosarcoma, which originates in the bone, is the most common bone sarcoma, with serious pain [168,169,170,171,172,173,174]. The overexpression of regucalcin suppressed the growth of human osteosarcoma Saos-2 cells by inhibiting several signaling pathways [98,175,176].

Ovarian cancer, which is a gynecological malignancy, shows an average five-year survival rate for tumor patients [177,178,179,180,181,182,183,184,185,186]. The overexpression of regucalcin suppressed colony formation and proliferation with the independent mechanism of cell death in SK-OV-3 human ovarian cancer cells in vitro [99], which are resistant to anticancer drugs [187]. The overexpression of regucalcin blocked the stimulatory effects of EGF on the proliferation of SK-OV-3 cells [99].

As described above, lower expression of regucalcin in human tumor tissue is associated with findings in patients with various types of cancer, including liver, pancreatic, breast, prostate, kidney, and colon cancer. The overexpression of regucalcin has suppressed the growth of various human cancer cells in translational studies in vitro. Regucalcin may be a potential suppressor of human cancer. Regucalcin may also have potential in patients with other types of cancer.

## 5. Extracellular Regucalcin Suppresses Human Cancer Cell Growth

Regucalcin is found in the serum, suggesting that extracellular regucalcin plays a role in the regulation of the function of several tissues and cells. Regucalcin is expressed in various organ cells in humans and animals, although its expression is remarkable in the liver and kidneys [187]. The physiological levels of regucalcin are approximately 1 nM in human serum [188]. Regucalcin is a secreted protein in saliva [189,190], although its functional role is not fully understood. Interestingly, isolated regucalcin binds to plasma membranes and increases (Ca^2+^-Mg^2+^)-adenosine triphosphatase activity, a Ca^2+^ pump enzyme [191], suggesting that extracellular regucalcin plays a role in regulating cell function. There has been increasing evidence that extracellular regucalcin inhibits the proliferation of several cancer cells, including liver, pancreatic, breast, and ovarian cancer, osteosarcoma, and glioblastoma. Extracellular regucalcin may play a potential role in the cancer microenvironment, representing a novel tool for cancer therapy.

### 5.1. Liver Cancer Cells

Regucalcin is released in the serum of human subjects with hepatitis [188,192,193], suggesting that it is a potential biomarker for the detection of hepatitis. The serum regucalcin concentration in all patients of 42 individuals diagnosed with liver disease was found at higher levels as compared with that in normal subjects without hepatitis [188]. In addition, the serum regucalcin levels of 33 healthy controls were compared with those of 47 chronic hepatitis B patients and 91 hepatitis B virus-related acute-on-chronic liver failure patients [192]. The serum regucalcin concentrations in the chronic hepatitis B patients and hepatitis B virus-related acute-on-chronic liver failure patients were extensively higher than those in the healthy controls [192,193]. Also, cases of hepatocellular carcinoma (HCC) are associated with chronic viral infections, including hepatitis B or hepatitis C [194,195,196,197,198]. Serum regucalcin may have clinical significance as a biomarker in human liver cancer. Regucalcin may be an HCC-associated antigen. Significant results were seen in alpha-fetoprotein (AFP)-negative patients [198]. These studies suggested that serum anti-regucalcin antibodies may be a novel biomarker for the diagnosis of HCC [199]. Serum regucalcin may play an important role in liver cancer.

Extracellular regucalcin has been shown to have repressive effects on cell growth in modeled human liver cancer HepG2 cells in vitro [200]. Physiological levels of extracellular regucalcin were found to suppress the colony formation of HepG2 cells in vitro [200]. The proliferation of liver cancer cells was suppressed through their culture with regucalcin (0.01–10 nM) in culture medium. Culturing with regucalcin had no effect on the death of the HepG2 cells in vitro. Interestingly, the addition of regucalcin suppressed the colony formation of cultured HepG2 cells. The inhibitory effects of extracellular regucalcin on cell growth were not attenuated through culture with various signaling inhibitors, including tumor necrosis factor-α (TNF-α), Bay K 8644, PD98059, staurosporine, wortmannin, 5,6-dichloro-1-β-D-ribofuranosyl benzimidazole (DRB), and gemcitabine, which suppressed liver cancer cell proliferation. The suppressive effects of extracellular regucalcin on liver cancer cell proliferation are mediated through different signaling pathways in vitro, including signaling pathways linked to NF-κB, intracellular calcium, MAPK, protein kinase C, PI3 kinase/Akt, transcription, and nuclear function. Thus, extracellular regucalcin has been shown to have suppressive effects on the growth of human liver cancer cells.

### 5.2. Pancreatic Cancer Cells

Pancreatic ductal adenocarcinoma is a highly aggressive malignancy [201,202,203]. In terms of the available therapies. there are limited treatment options for it [201,202,203]. K-ras mutations are caused in pancreatic cancers [204,205,206,207]. Extracellular regucalcin has also been shown to block the proliferation of MiaPaCa-2 human pancreatic cancer cells in vitro [208]. MiaPaCa-2 cell proliferation was suppressed through culture with extracellular regucalcin (0.01–10 nM). This suppression was not affected by the presence of several regulators in signaling pathways, including PD98059, staurosporine, wortmannin, DRB, and gemcitabine [208]. Extracellular regucalcin (0.01–10 nM) did not cause apoptotic cell death in vitro [208]. Extracellular regucalcin may reveal inhibitory effects on pancreatic MiaPaCa-2 cell growth in vitro mediated by different signaling pathways in vitro.

### 5.3. Breast Cancer Cells

Extracellular regucalcin has been shown to block the growth of MDA-MB-231 human breast cancer cells [209]. Breast cancer is the most common form of malignancy and the leading cause of cancer deaths among women in the United States. This malignancy tends to metastasize to bone [118,119,120,121,122,123,124,125,126], causing distressing pathological fractures, pain, and hypercalcemia. Cancer cell invasion into the bone tissues leads to the activation of the osteoclasts and osteoblasts. Bisphosphonates or denosumab is the standard-of-care treatment for breast cancer patients with bone metastases [123,124,125,126]. MDA-MB-231 cell proliferation is suppressed through culture with extracellular regucalcin (0.01–10 nM), which affects various signaling pathways [209]. The inhibitory effects of extracellular regucalcin on cell proliferation were not independent of apoptotic cell death of the MDA-MB-231 cells [209].

### 5.4. Prostate Cancer Cells

Prostate cancer is a malignancy that often spreads to the bones [112,113,114,115], leading to tumor growth or disease recurrence associated with serious complications like pain, fractures, spinal cord compression, and bone marrow suppression [210,211,212,213,214,215,216]. Culturing with extracellular regucalcin (0.1–10 nM) at serum levels suppressed cancer cell growth and the metastatic activity, including the migration, invasion, and adhesion, of PC-3 and DU-145 human prostate cancer cells in vitro, and it did not affect cell death [217]. The suppressive effects of extracellular regucalcin on proliferation were not caused by culture with various inhibitors of the cell cycle, the intracellular signaling process, and transcriptional activity, suggesting that signals from extracellular regucalcin are transmitted to block cell growth. Furthermore, extracellular regucalcin (0.1, 1, or 10 nM) inhibited the migration, invasion, and adhesion of PC-3 and DU-145 cells. Mechanistically, extracellular regucalcin decreased the levels of several signaling-related proteins, including Ras, phosphatidylinositol-3 kinase, mitogen-activated protein kinase, mTOR, RSK-2, caveolin-1, and integrin β1, in PC-3 cells. Thus, extracellular regucalcin may play a suppressive role in growth, migration, invasion, and adhesion, which are involved in the metastatic activity of human prostate cancer cells, via affecting various signaling pathways, providing a new strategy for preventing metastatic prostate cancer using exogenous regucalcin.

### 5.5. Ovarian Cancer Cells

Ovarian cancer has the highest mortality rate among gynecological malignancies, with an average five-year survival rate for tumor patients [177,178,179]. It is a complex and heterogeneous malignancy. Extracellular regucalcin was found to suppress the proliferation of SK-OV-3 human ovarian cancer cells independently of cell death in vitro [99]. SK-OV-3 cell proliferation was increased through culture with EGF [99], while it was blocked through culture with extracellular regucalcin (0.01–10 nM) [99]. Extracellular regucalcin did not decrease the EGF receptor protein levels in the SK-OV-3 cells [99]. Extracellular regucalcin may block EGF from binding to its receptors, located on cell plasma membranes. Extracellular regucalcin may suppress cell proliferation by targeting several signaling pathways associated with EGF-signaling-related molecules, including Ras, Akt, mitogen-activating protein kinase, NF-κB p65, β-catenin, and STAT3, the tumor suppressors p53 and Rb, and the cell cycle inhibitor p21. This may be accomplished by targeting specific proteins in a mechanistic manner.

### 5.6. Osteosarcoma Cells

Osteosarcoma originates in the bone, and there have been few advances in terms of survival and treatment of metastatic disease [168,169,170,171]. Chondrosarcoma is the most common bone sarcoma in human adults [174,175]. Pain is the most common presenting symptom in patients with bone tumors [186]. Primary tumors in osteosarcoma are surgically resected [178,179,180,181,182,183,184,185,186,187]. The involvement of regucalcin in human osteosarcoma has been investigated using Saos-2 human osteosarcoma cells in vitro [98]. Culturing with exogenous regucalcin (1 and 10 nM) resulted in decreased colony formation and proliferation of Saos-2 cells, although it did not cause Saos-2 cell death [98]. Mechanistically, extracellular regucalcin decreased the expression of Ras, PI3K, Akt, MAP kinase, phosphor-MAP kinase, STAT3, NF-κB p65, and β-catenin, which are linked to the intracellular signaling pathways. In addition, culture with extracellular regucalcin increased cell proliferation and the suppressor p21. Exogenous regucalcin may be beneficial in the treatment of osteosarcoma associated with increased cell proliferation.

### 5.7. Glioblastoma Cells

Glioblastoma is the most common malignant brain tumor in adults [218,219,220]. This tumor is aggressive and the most lethal. Trials on improving the outcomes of patients with this tumor remain critical. There are no effective treatments for malignant glioma [218,219,220]. Glioblastoma is characterized by the ligand-independent overexpression of EGF receptors [221,222,223,224,225]. EGF receptor signaling can promote tumorigenesis by increasing cell proliferation and metastatic activity and by inhibiting apoptosis of the cancer cells. Interestingly, culturing with extracellular regucalcin suppressed cancer cell growth, including cell proliferation and colony formation. Culturing with regucalcin (0.01–10 nM) suppressed EGF-enhanced cell proliferation, independent of alterations in the EGF receptor levels and cell death of the glioblastoma cells. The inhibitory effects of extracellular regucalcin on cell proliferation were not attenuated by treatment with various intracellular signaling inhibitors, including genistein, a tyrosine kinase inhibitor, and an MAPK inhibitor. Mechanistically, the regucalcin treatment decreased PI3-kinase 100α, Akt, MAPK, phosphor-MAPK, and mTOR, linked to cell growth promotion. Notably, regucalcin treatment inhibited the metastatic activity, including the adhesion, migration, and invasion, of cancer cells. Thus, extracellular regucalcin may inhibit the activity of human glioblastoma cells, suggesting its suppressive role in the cancer microenvironment.

As mentioned above, there are growing investigations indicating that extracellular regucalcin suppresses the proliferation and metastatic activity of several types of human cancer cells in vitro, including in liver, pancreatic, breast, and prostate cancer; osteosarcoma; ovarian cancer, and glioblastoma, without affecting cell death. Extracellular regucalcin may affect other types of cancer cells. Extracellular regucalcin produced in the tissues is likely to play an important role in suppressing the growth of cancer cells. Extracellular regucalcin may be a cytokine suppressing cancer cell growth.

**Figure 1:**
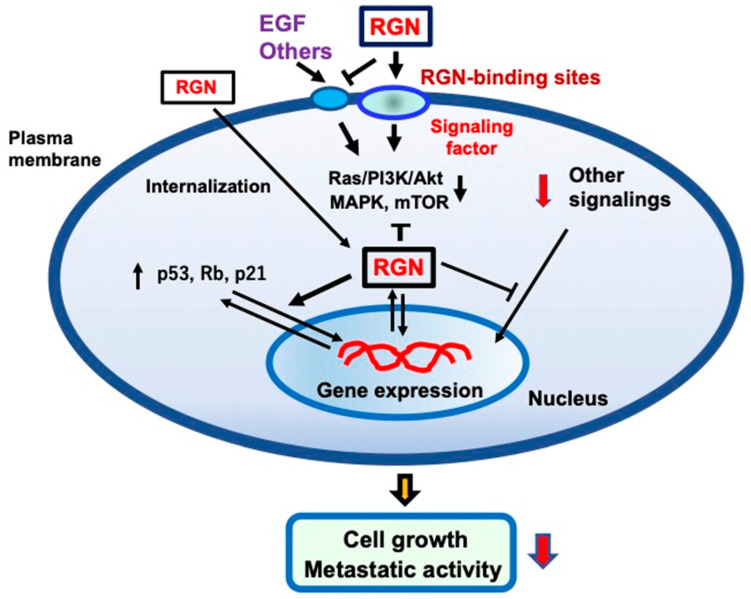
The mechanistic hypothesis on how extracellular regucalcin (RGN) exerts anticancer effects on human cancer cells. Extracellular RGN suppresses cell proliferation and colony formation independently from apoptotic cell death. Extracellular RGN binds to the plasma membranes and regulates the signal transduction activity in the plasma membranes. In particular, RGN may antagonize the binding of various peptide hormones and cytokines, including epidermal growth factor (EGF). This can lead to the inhibition of the EGF signaling pathways, including Ras/PI3K/Akt/MAPK and mTOR pathways. Extracellular RGN can be internalized into the cells and regulate intracellular signaling systems. In addition, intracellular regucalcin may regulate nuclear function, leading to increased levels of Rb, p53, and p21, which are tumor suppressors, in cancer cells. Thus, extracellular RGN may have anticancer effects in several types of cancer. Abbreviations: RGN; regucalcin, EGF; epidermal growth factor.

The putative mechanism through which extracellular regucalcin suppresses cancer cell growth is shown in Figure 1. Whether a specific binding site for regucalcin exists on cell plasma membranes has not yet been identified, although regucalcin can bind to the plasma membranes [191]. Extracellular regucalcin being bound to the plasma membranes of cancer cells may generate a certain factor that suppresses the intracellular signaling pathways linked to nuclear transcription in cancer cells. In addition, regucalcin binding to the cell surface may cause cell internalization. Internalized regucalcin may affect various cell signaling pathways, resulting in the suppression of cell proliferation. Extracellular regucalcin can block the binding of EGF to its receptor and inhibit its related signaling pathways. Extracellular regucalcin being increased in the microenvironment may prevent cancer cell growth. In addition, regucalcin in the microenvironment may inhibit the metastatic activity, including the adhesion, invasion, and migration, of cancer cells. Circulating regucalcin, which is altered under various physiological and pathophysiological conditions, may play a potential role in preventing and suppressing carcinogens and cancer cell metastasis in the cancer microenvironment. Injections of exogenous regucalcin may be a useful tool in cancer treatment, although many clinical trials would be needed.

## 6. Extracellular Regucalcin Levels Are Attenuated by Several Factors

**Figure 2:**
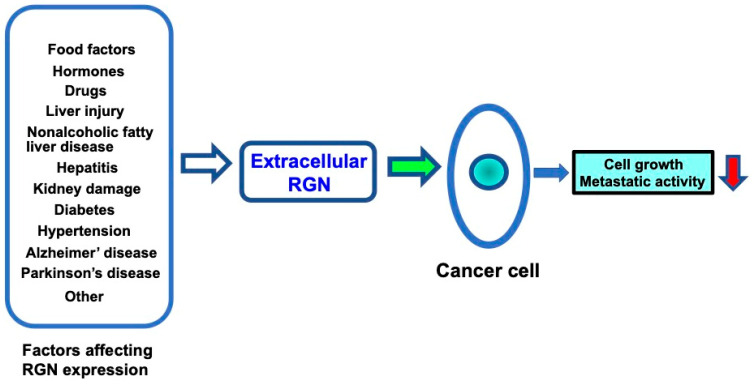
Altered extracellular regucalcin in serum and fluids in the cancer cell microenvironment affects cancer cell activity. Regucalcin gene expression and regucalcin levels in tissues and cells are altered by several factors involved in physiological and pathophysiological conditions. Altered extracellular regucalcin may affect cancer cell activity. Reduced extracellular regucalcin may enhance cancer cell activity, including cell growth and metastasis, leading to the progression of carcinogenesis, while increased extracellular regucalcin may inhibit cancer cell activity. Abbreviations: RGN = regucalcin.

Several factors may influence extracellular regucalcin levels, as shown in Figure 2. Regucalcin is expressed in several tissues [187]. In particular, the expression of regucalcin is highly expressed in the liver and kidneys. The regucalcin levels in the liver are estimated to be 80 and 52 μM in male and female rats, respectively [187]. Regucalcin expression in the tissue is altered by liver and kidney damage [45], suggesting the role of regucalcin in the development of these diseases. Altered serum regucalcin may be of clinical significance as a biomarker in diseases [45]. In particular, changes in serum regucalcin may influence the extracellular regucalcin levels in the cancer cell microenvironment. Reduced serum regucalcin may affect the extracellular regucalcin levels and contribute to cancer progression in the cancer microenvironment.

Several chemicals and drugs are known to affect the regucalcin expression in the liver. Hepatic regucalcin mRNA expression is suppressed after a single oral dose of carbon tetrachloride (CCl_4_), which is known to be a chemical inducer of liver injury [44]. The serum regucalcin concentrations were increased after the oral administration of CCl_4_ in rats, suggesting that regucalcin is released from the liver into the serum during acute liver injury [44]. Reduced levels of regucalcin in the liver tissue environment may be involved in the progression of liver injury and carcinogenesis because regucalcin plays a role in suppressing liver cancer cells. Phenobarbital is an inducer of the enzymes that are involved in the chemical metabolism of many drugs and endogenous steroids [226]. The hepatic regucalcin mRNA expression and regucalcin levels in rats were significantly reduced by the administration of phenobarbital [227]. The administration of streptozotocin, which induces type I diabetes, with impairment of the insulin secretion in the pancreatic cells, decreases the regucalcin levels in rat livers [45]. Diabetic states may cause a decrease in the regucalcin levels in the cancer cell microenvironment. Hepatic regucalcin levels were also reduced by ethanol administration in rats [45]. Reduced regucalcin may influence its levels in the tissue fluids of the microenvironment, leading to the development of carcinogenesis. In addition, regucalcin is increased in the serum of hepatitis patients with chronic liver disease [188,199,228]. Thus, extracellular regucalcin may be affected by liver disease. Altered extracellular regucalcin may contribute to the progression of carcinogenesis and enhance the metastatic activity of cancer cells.

Regucalcin gene expression is suppressed by the development of hypertensive states and drug-induced renal damage [229,230]. Regucalcin mRNA expression is decreased in the renal cortex of spontaneously hypertensive rats as compared to control (Wistar–Kyoto) rats [231,232], suggesting the involvement of renal hypertension. Several drugs and other chemicals are known to have side effects that cause kidney damage [233,234,235,236]. Renal regucalcin expression is suppressed by drugs that induce renal damage. Cisplatin, a nephrotoxic antitumor drug [233], and cephaloridine, a nephrotoxic cephalosporin antibiotic drug [234], induce a change in the thiol status in the renal cortex. The regucalcin mRNA expression in the renal cortex is markedly suppressed after the administration of cisplatin or cephaloridine [237]. Chemical-administration-induced suppression of the expression of regucalcin mRNA in the kidneys may be implicated in in the development of renal damage [237]. Ochratoxin A (OTA), a naturally occurring mycotoxin, is nephrotoxic in all animal species and is considered to be a potent renal carcinogen, and regucalcin is strongly suppressed by its administration [238]. In addition, prolonged exposure to aristolochic acid is associated with the development of kidney damage and downregulates the expression of regucalcin in the kidneys [239]. Reduced regucalcin in the kidneys may influence the levels of regucalcin in the tissue fluids of the cancer microenvironment, leading to the development of carcinogenesis and increased metastatic activity.

In addition, diabetic nephropathy (DN) is a major complication of diabetes mellitus and the most common cause of end-stage renal disease [240]. Regucalcin is strongly downregulated in kidney tissue in DN compared with healthy controls [240]. Interestingly, regucalcin was detected in exosomes isolated from the urine of healthy donors but not in urine from patients with kidney disease [240]. The expression of regucalcin is reduced in the kidney tissue in DN in urine-isolated exosomes. Exosomes play a role as a critical communicator between the tumor microenvironment and cancer [241]. Thus, the downregulation of regucalcin in the kidneys may lead to renal disease and carcinogenesis with altered extracellular regucalcin. 

Neuronal Ca^2+^ signaling has been implicated in the mechanisms of neuronal plasticity, such as long-term potentiation, which is thought to play an important role in learning and memory. Regucalcin is expressed in the neurons in rat brains and decreases with age in the cerebral cortex and hippocampus of the brain [242,243]. Regucalcin was found to have an inhibitory effect on calcium signaling in the neurons in rats’ brains [244]. The inhibitory effects of regucalcin are attenuated with age [244]. Interestingly, regucalcin has been implicated in Parkinson’s and Alzheimer’s disease [244,245]. A total of 2495 proteins were identified in a proteomic analysis, of which 87 proteins, including regucalcin, were differentially expressed in the locus coeruleus of Parkinson’s patients compared to controls [244]. These proteins are implicated in the pathogenesis of Parkinson’s disease [244]. In addition, the neurofibrillary tangles formed by the accumulation of abnormal tau filaments are implicated in brain degeneration in Alzheimer’s disease [245]. Novel biomarker candidates for the early diagnosis of Alzheimer’s disease have been investigated using a tau transgenic mouse model [245]. Regucalcin has been found to be decreased in the plasma of tau transgenic mice [245], suggesting its significance as a candidate biomarker in the early diagnosis of Alzheimer’s disease. Thus, regucalcin may play an important role in the detection of brain diseases, including Parkinson’s and Alzheimer’s diseases. Downregulation of the regucalcin expression in the brain tissues may play a role in the development of brain cell carcinogenesis. Reduced levels of regucalcin in the brain tissue and its microenvironment may therefore contribute to the development of brain tumors.

As described above, the extracellular regucalcin levels may be altered by several factors under physiological and pathophysiological states. Changes in extracellular regucalcin may affect the microenvironment of cancer cells, leading to tumor progression and increased metastatic activity.

## 7. Conclusions and Perspectives

Regucalcin plays a critical role as a regulator of cell signaling in maintaining cell homeostasis by suppressing enhanced cell growth and preventing stimulated apoptotic cell death [21]. Regucalcin expression has been shown to be altered in the pathophysiological states of several diseases [21,22,23,24,25,26,27,28,29]. As discussed in this review, intracellular regucalcin plays a critical role as a novel suppressor in human cancer patients. Regucalcin levels are significantly decreased in the tissues of cancer patients, including in liver, pancreas, colon, lung, kidney, breast, prostate, and cervical cancer and melanoma, and may shorten the survival of cancer patients [34]. Further clinical trials may show the involvement of regucalcin in other human cancers. Carcinogenesis may be prevented by increasing the expression of regucalcin in the tumor tissue. Delivering the regucalcin gene to tumor tissues may be a useful tool in cancer therapy.

Tissue regucalcin is released into the serum during physiological and pathophysiological conditions, and the secreted regucalcin may have a role as a regulatory factor in the fluid microenvironment. Recent studies have shown that extracellular regucalcin suppresses the growth of several types of human cancer cells, including liver cancer, pancreatic cancer, breast cancer, prostate cancer, ovarian cancer, osteosarcoma cells, and glioblastoma, in vitro. The role of extracellular regucalcin remains to be elucidated in other types of cancer cells. Extracellular regucalcin may play a critical role in suppressing cancer cell growth in the cancer microenvironment. Specifically, extracellular regucalcin inhibits metastatic activity, including the adhesion, invasion, and migration of human cancer cells, in vitro [217]. Extracellular regucalcin has been proposed to play an important role in the prevention of cancer development. However, the detailed mechanism by which extracellular regucalcin suppresses cancer cell growth remains to be investigated.

Extracellular regucalcin may be a novel biomarker for the diagnosis and treatment of several cancers. Extracellular regucalcin may have potential clinical significance. Regucalcin expression has been shown to be regulated by physiological factors, including dietary factors, hormones, and its related intracellular signaling and transcription factors [20,24,42]. In addition, the regucalcin expression in different tissues has been shown to be downregulated by pathophysiologic conditions and pathophysiologic factors, including aging, drug intake, environmental factors, and various diseases states. As shown in Figure 2, altered expression of regucalcin may affect the levels of extracellular regucalcin. The metastasis and growth of cancer cells may be accelerated by alterations in several factors in the cancer microenvironment. Extracellular regucalcin may contribute to the prevention and suppression of carcinogenesis and cancer metastasis.

Exogenous regucalcin can be delivered into the cancer microenvironment. Treatment with exogenous regucalcin has a clinical role in suppressing tumor development and may be useful as a new tool in cancer control. However, the clinical application of this therapy may face several challenges. Clinical research may be needed to establish the usefulness of extracellular regucalcin in suppressing human cancer.

## Data Availability

The datasets used in this study are available from the corresponding author upon reasonable request.
